# Supplementary material for: Machine learning optimization of candidate antibody yields highly diverse sub-nanomolar affinity antibody libraries
Source: Nat Commun. 2023 Jun 12;14:3454. doi: 10.1038/s41467-023-39022-2 (PMC10258481; doi:10.1038/s41467-023-39022-2)
Supplement: Supplementary file 1 — Supplementary Information [file 41467_2023_39022_MOESM1_ESM.pdf]

Supplementary Information for

Machine Learning Optimization of Candidate Antibody Yields Highly Diverse Sub-nanomolar Affinity Antibody Libraries

Lin Li[1*], Esther Gupta[1], John Spaeth[1], Leslie Shing[1], Rafael Jaimes[1], Emily Engelhart[2], Randolph Lopez[2], Rajmonda S. Caceres[1,†], Tristan Bepler[3,4,†] and Matthew E. Walsh[1,5,†]

[1]Massachusetts Institute of Technology Lincoln Laboratory, Lexington, MA, USA

[2]A-Alpha Bio, Inc., Seattle, WA, USA

[3]Research Laboratory of Electronics, Massachusetts Institute of Technology, Cambridge, MA, USA

[4]Present address: Simons Electron Microscopy Center, New York Structural Biology Center, New York, NY, USA

[5]Present address: Johns Hopkins Bloomberg School of Public Health, Baltimore, MD, USA

[†]These authors contributed equally to this work.

E-mail: Lin.Li@LL.MIT.EDU

Table of Contents

SUPPLEMENTARY TABLES **3**

Supplementary Table 1. Target sequence and candidate scFv sequences (CDRs in bold). 3

Supplementary Table 2. Distribution of mutations within each initial scFv library. 3

Supplementary Table 3. Train, validation and test splits for protein/antibody language model training. 4

Supplementary Table 4. Percentage incorporation of Ab-14-H designs by library. 4

Supplementary Table 5. Percentage incorporation of Ab-14-L designs by library. 4

Supplementary Table 6. The best heavy chain sequences by library (CDRs in bold) 5

Supplementary Table 7. The best light chain sequences by library (CDRs in bold) 6

Supplementary Table 8. Ranking of Libraries Using Estimated Percent of Success 6

Supplementary Table 9. Regression models used in the ensemble-based fitness model. 7

SUPPLEMENTARY FIGURES **8**

Supplementary Fig. 1 Empirical binding distribution of training data and designed scFvs. 8

Supplementary Fig. 2 Sequence-to-affinity model evaluation on the hold-out test data. 9

Supplementary Fig. 3 Distribution of diversity metrics by library for Ab-14-H variants. 10

Supplementary Fig. 4 Distribution of diversity metrics by library for Ab-14-L variants. 11

Supplementary Fig. 5 Random mutation library vs PSSM library. 12

Supplementary Fig. 6 Heavy chain designs vs light chain designs. 13

Supplementary Fig. 7 Evaluation of proposed in silico metric for library performance prediction. 13

Supplementary Fig. 8 The estimated percent of success metric enables exploration of the tradeoffs between performance and diversity and informs library selection (Ab-14-H variant designs). 14

Supplementary Fig. 9 The estimated percent of success metric enables exploration of the tradeoffs between performance and diversity and informs library selection (Ab-14-L variant designs). 14

Supplementary Fig. 10 Evaluation of designing various antibody CDR regions. 15

Supplementary Fig. 11 Biophysical and sequence properties of heavy and light chain libraries. 15

Supplementary Fig. 12 Masked language modeling with BERT transformer. 16

Supplementary Tables

Supplementary Table 1. Target sequence and candidate scFv sequences (CDRs in bold). Three candidate scFvs (i.e., Ab-14, Ab-91, Ab-95) were first identified *in vitro* from a human-derived naïve phage-display campaign against the target peptide. Two heavy chains (H) and two light chains (L) from the three scFvs were selected for the generation of the initial scFv library. They are Ab-14-H, Ab-91-H, and Ab-14-L, Ab-95-L.

**Target**	PDVDLGDISGINAS
**Candidate Chains**	**ScFv Sequences**
Ab-14-H	EVQLVETGGGLVQPGGSLRLSCAAS**GFTLNSYGIS**WVRQAPGKGPEWVSV**IYSDGRRTFYGDSV**<br>KGRFTISRDTSTNTVYLQMNSLRVEDTAVYYCAK**GRAAGTFDS**WGQGTLVTVSS
Ab-14-L	DVVMTQSPESLAVSLGERATISC**KSSQSVLYESRNKNSVA**WYQQKAGQPPKLLIY**WASTRES**GV<br>PDRFSGSGSGTDFTLTISSLQAEDAAVYYC**QQYHRLPLS**FGGGTKVEIK
Ab-91-H	EVQLVESGGGLVQPGRSLRLSCAAS**GFTFDDYAMH**WVRQAPGKGLEWVS**GISWNSGSIGYADSV**<br>**K**GRFTISRDNAENSLYLQMNSLRAEDTALYYCAK**VGRGGGYFDY**WGQGTLVTVSS
Ab-91-L	QAVLTQPSSLSASPGASVSLTC**TLRSGINVGTYRIY**WYQQKPGSPPQYLLR**YKSDSDKQQGSGV**<br>PSRFSGSKDASANAGILLISGLQSEDEADYYC**MIWHSSAWV**FGGGTKLTVL
Ab-95-H	EVQLVESGAEVKKPGASVKVSCKAS**GYTFTSYGIS**WVRQAPGQGLEWMGW**ISAYNGNTNYAQ**KL<br>QGRVTMTTDTSTSTAYMELRSLRSDDTAVYYCAR**VGRGVIDH**WGQGTLVTVSS
Ab-95-L	SSELTQDPAVSVALGQTVRITC**EGDSLRYYYAN**WYQQKPGQAPILVIY**GKNNRPS**GIADRFSGS<br>NSGDTSSLIITGAQAEDEADYYC**SSRDSSGFQVF**FGAGTKLTVL

Supplementary Table 2. Distribution of mutations within each initial scFv library. Given the candidate scFv, *k*-point mutations, where *k= 1, 2,* and *3*, were designed across all CDRs of each chain (heavy or light). Point mutations were limited to amino acid substitutions. The numbers before and after the slash line represent the number of variants present in the experimental measurements and the total numbers of variant designs, respectively. The missing sequences are because they were unsuccessfully mapped in the haploid step and thus had no binding affinity data available.

Library	k=1	k=2	k=3
Ab-91-H Variants	521/684	3,131/4,141	18,820/25,075
Ab-14-H Variants	594/665	3,671/4,089	22,188/25,146
Ab-14-L Variants	552/627	3,491/3,982	22,180/25,291
Ab-95-L Variants	548/551	3,743/3,755	25,526/25,594

Supplementary Table 3. Train, validation and test splits for protein/antibody language model training. Values in the parenthesis indicate the number of heavy-light sequence pairs in OAS.

Datasets	Train	Validation	Test
Pfam	32,593,668	1,715,454	44,311
OAS Heavy Chains	172,524,747	47,603,347	51,043,837
OAS Light Chains	70,059,824	364,332	414,635
OAS Heavy-Light Chains	242,612,962 (28,391)	47,972,437 (4758)	51,459,204 (732)

Supplementary Table 4. Percentage incorporation of Ab-14-H designs by library. Some sequences are absent from the resulting experimental dataset because they were unsuccessfully mapped in the haploid step and thus had no binding affinity data available.

Library	No. Sequences Designed	No. Sequences Present	Overall % Present
Ensemble-HC	6,000	5,344	89%
Ensemble-Gen	6,000	5,310	89%
Ensemble-Gibbs	6,000	4,879	81%
GP-HC	6,000	5,152	86%
GP-Gen	6,000	5,313	89%
GP-Gibbs	6,000	5,284	88%
PSSM	7,748	6,510	84%

Supplementary Table 5. Percentage incorporation of Ab-14-L designs by library. Some sequences are absent from the resulting experimental dataset because they were unsuccessfully mapped in the haploid step and thus had no binding affinity data available.

Library	No. Sequences Designed	No. Sequences Present	Overall % Present
Ensemble-HC	6,000	5,962	99%
Ensemble-Gen	6,000	5,960	99%
Ensemble-Gibbs	6,000	5,950	99%
GP-HC	6,000	5,965	99%
GP-Gen	6,000	5,989	100%
GP-Gibbs	6,000	5,987	100%
PSSM	8,257	8,188	99%

Supplementary Table 6. The best heavy chain sequences by library (CDRs in bold)

Libraries	Best Ab-14-H Variant
Random Mutations	EVQLVETGGGLVQPGGSLRLSCAAS**GFTLNQYGIS**WVRQAPGKGPEWVSV**IYSDGIRTFYSDSV**KG<br>RFTISRDTSTNTVYLQMNSLRVEDTAVYYCAK**GRAAPFFDS**WGQGTLVTVSS
PSSM	EVQLVETGGGLVQPGGSLRLSCAAS**GFTLNEYGIS**WVRQAPGKGPEWVSV**IYADGRRTFYADSV**KG<br>RFTISRDTSTNTVYLQMNSLRVEDTAVYYCAK**GRAAGTFDV**WGQGTLVTVSS
GP-HC	EVQLVETGGGLVQPGGSLRLSCAAS**GFTLNEYGIS**WVRQAPGKGPEWVSV**IYSDGRRTFYSDSV**KG<br>RFTISRDTSTNTVYLQMNSLRVEDTAVYYCAK**GRAAGTFDI**WGQGTLVTVSS
GP-Gen	EVQLVETGGGLVQPGGSLRLSCAAS**GFSLNEYGIS**WVRQAPGKGPEWVSV**IYSDGRRTFYGDSV**KG<br>RFTISRDTSTNTVYLQMNSLRVEDTAVYYCAK**GQAAGTFDF**WGQGTLVTVSS
GP-Gibbs	EVQLVETGGGLVQPGGSLRLSCAAS**GFSLNEYGIS**WVRQAPGKGPEWVSV**IYSDGRRTFYGDSV**KG<br>RFTISRDTSTNTVYLQMNSLRVEDTAVYYCAK**GNAAGTFDQ**WGQGTLVTVSS
En-HC	EVQLVETGGGLVQPGGSLRLSCAAS**GFDLNEYGIS**WVRQAPGKGPEWVSV**IYADGRRTFYTDSV**KG<br>RFTISRDTSTNTVYLQMNSLRVEDTAVYYCAK**GEVAGTFDG**WGQGTLVTVSS
En-Gen	EVQLVETGGGLVQPGGSLRLSCAAS**GFDLNEYGIS**WVRQAPGKGPEWVSV**IYADGSRKAYADSV**KG<br>RFTISRDTSTNTVYLQMNSLRVEDTAVYYCAK**GNNAGTFDV**WGQGTLVTVSS
En-Gibbs	EVQLVETGGGLVQPGGSLRLSCAAS**EFDIQEYGIS**WVRQAPGKGPEWVSV**IYADGKREAYKDKF**KG<br>RFTISRDTSTNTVYLQMNSLRVEDTAVYYCAK**GQVAGTFDA**WGQGTLVTVSS

Supplementary Table 7. The best light chain sequences by library (CDRs in bold)

Libraries	Best Ab-14-L Variant
Random Mutations	DVVMTQSPESLAVSLGERATISC**KSSQSVLYESRNKNSVA**WYQQKAGQPPKLLIY**WASTRES**GVPD RFSGSGSGTDFTLTISSLQAEDAAVYYC**QQYHRLPLS**FGGGTKVEIK<br>DVVMTQSPESLAVSLGERATISCKQSQEVLFESRNKNSVAWYQQKAGQPPKLLIY**DASTRES**GVPD RFSGSGSGTDFTLTISSLQAEDAAVYYC**QQYHRLPLS**FGGGTKVEIK
PSSM	DVVMTQSPESLAVSLGERATISC**KLSQSVLYESRNKNSVA**WYQQKAGQPPKLLIY**DASLRES**GVPD RFSGSGSGTDFTLTISSLQAEDAAVYYC**QQYHRLPLS**FGGGTKVEIK
GP-HC	DVVMTQSPESLAVSLGERATISC**KSSQSVLYESGNKNSVA**WYQQKAGQPPKLLIY**DASTRED**GVPD RFSGSGSGTDFTLTISSLQAEDAAVYYC**QQYHRLPLS**FGGGTKVEIK
GP-Gen	DVVMTQSPESLAVSLGERATISC**KVQQSVLYESRNKNSVA**WYQQKAGQPPKLLIY**GASTRES**GVPD RFSGSGSGTDFTLTISSLQAEDAAVYYC**QQYHRLPLS**FGGGTKVEIK
GP-Gibbs	DVVMTQSPESLAVSLGERATISC**KLMQEDEYQSRNPNSVA**WYQQKAGQPPKLLIY**HASERES**GVPD RFSGSGSGTDFTLTISSLQAEDAAVYYC**QQYHRLPLS**FGGGTKVEIK
En-HC	DVVMTQSPESLAVSLGERATISC**MISESVMYESRNRNNVA**WYQQKAGQPPKLLIY**DHSTRED**GVPD RFSGSGSGTDFTLTISSLQAEDAAVYYC**QCYDRLPLS**FGGGTKVEIK
En-Gen	DVVMTQSPESLAVSLGERATISC**QISGIQGHMSTIKNNVA**WYQQKAGQPPKLLIY**EMVTRAN**GVPD RFSGSGSGTDFTLTISSLQAEDAAVYYC**QQYERLPLS**FGGGTKVEIK
En-Gibbs	DVVMTQSPESLAVSLGERATISC**NMVEDEAGDQKNSGNIA**WYQQKAGQPPKLLIY**SVDQRED**GVPD RFSGSGSGTDFTLTISSLQAEDAAVYYC**QQYQKLPLM**FGGGTKVEIK

Supplementary Table 8. Ranking of Libraries Using Estimated Percent of Success

Ensemble Models					GP Models				
**Method**	**Ab-14-H Variant Libraries**		**Ab-14-L Variant Libraries**		**Method**	**Ab-14-H Variant Libraries**		**Ab-14-L Variant Libraries**	
	**Rank (Predicted)**	**Rank (Actual)**	**Rank (Predicted)**	**Rank (Actual)**		**Rank (Predicted)**	**Rank (Actual)**	**Rank (Predicted)**	**Rank (Actual)**
PSSM	4	3	4	4	PSSM	4	4	4	4
En-HC	2	2	1	1	GP-HC	3	3	2	3
En-GA	1	1	2	3	GP-GA	1	1	1	1
En-Gibbs	3	4	3	2	GP-Gibbs	2	2	3	2
	Rank Correlation: 0.8		Rank Correlation: 0.8			Rank Correlation: 1		Rank Correlation: 0.8	

Supplementary Table 9. Regression models used in the ensemble-based fitness model.

Name	Base Model	Loss Function	Missing Values
pfam_drop_l1	Pfam language model	MAE	Drop
pfam_drop_mse	Pfam language model	MSE	Drop
pfam_median_l1	Pfam language model	MAE	Impute with median value
pfam_median_mse	Pfam language model	MSE	Impute with median value
heavy_drop_l1	Heavy-chain model	MAE	Drop
heavy_drop_mse	Heavy-chain model	MSE	Drop
heavy_median_l1	Heavy-chain model	MAE	Impute with median value
heavy_median_mse	Heavy-chain model	MSE	Impute with median value
light_drop_l1	Light-chain model	MAE	Drop
light_drop_mse	Light-chain model	MSE	Drop
light_median_l1	Light-chain model	MAE	Impute with median value
light_median_mse	Light-chain model	MSE	Impute with median value
paired_drop_l1	Paired model	MAE	Drop
paired_drop_mse	Paired model	MSE	Drop
paired_median_l1	Paired model	MAE	Impute with median value
paired_median_mse	Paired model	MSE	Impute with median value

Supplementary Figures

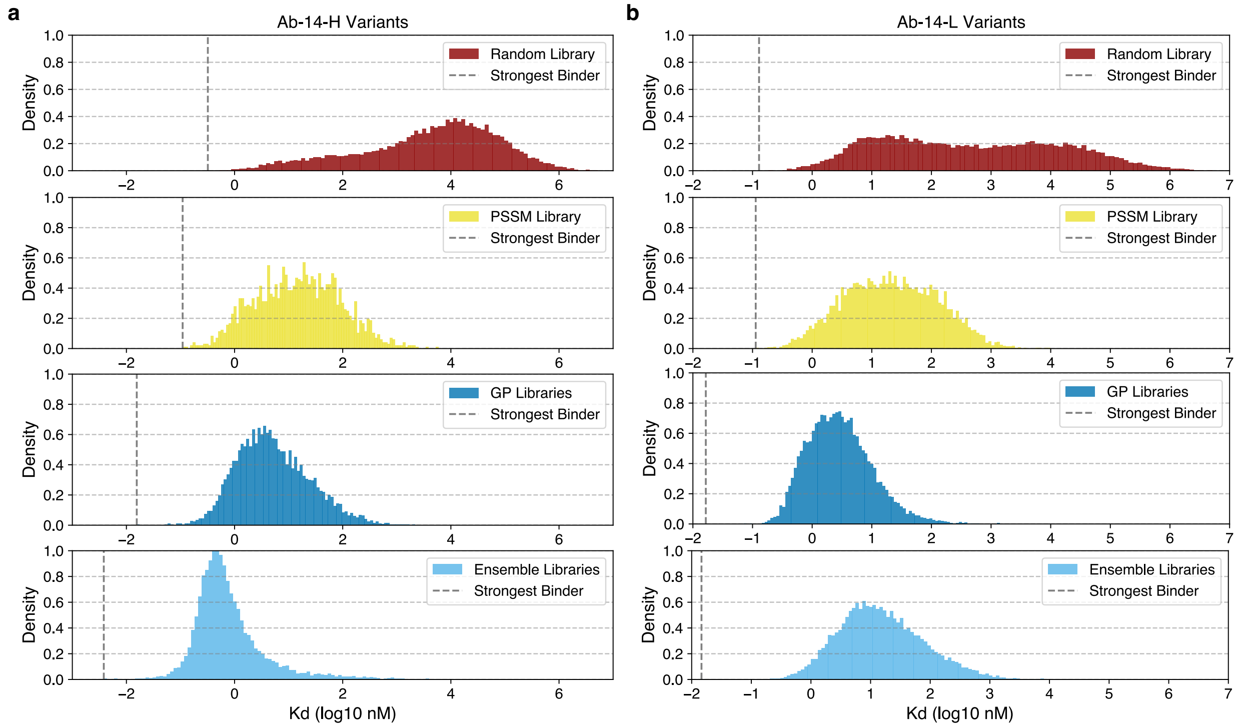

Supplementary Fig. 1 Empirical binding distribution of training data and designed scFvs. Average affinity is used for each sequence. For the training data (random library), the average value was computed for sequences with at least 1 (out of 3) empirically measure binding affinities. For the PSSM, GP and Ensemble libraries, the average value was computed for sequences with at least 3 (out of 6) empirical binding affinities. (**a**) Distributions of Ab-14 heavy-chain designs and the corresponding best binder. (**b**) Distributions of Ab-14 light-chain designs and the corresponding best binder. The top row in both plots shows the distribution of the training data (random library) and the rest of the rows are the distributions of the designed sequences using the various methods. Note how the ML designs are more tightly concentrated around higher affinity values (lower Kds) when compared to the training distributions of both variants. Source data are provided as a Source Data file.

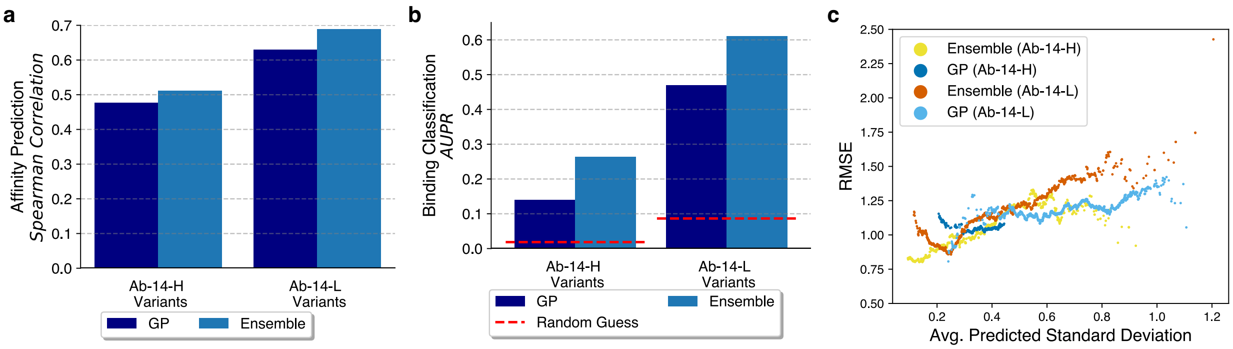

Supplementary Fig. 2 Sequence-to-affinity model evaluation on the hold-out test data. (**a**) Regression performance; the ensemble model is more predictive than the GP model. (**b**) We define an additional model evaluation task: the model's ability to classify strong binders and weak binders. Sequences are labeled as strong binders if the empirically measured affinities are stronger (lower Kd values) than the initial candidate sequence and weak binders if the empirically measured affinities are weaker (higher Kd values) than the candidate sequence. The random guess computes the ratio of the number of strong binders to the total number of sequences in the hold-out test data. Area under the precision-recall curve (AUPR) is used to evaluate the binding classification task because it is tailored for the detection of rare events as suggested by random guess values. To compute the AUPR, we labeled all strong binders with a ground truth label '1' and weak binders with a ground truth label '0'. Since the lower the Kd value, the stronger the binder, we first negated all model's predicted Kd values (thus the higher the predicted value, the stronger the model predicts the binder to be) and then computed the precision-recall (PR) curve from which the AUPR was calculated. The PR curve computes precision-recall pairs for different threshold values and the AUPR estimates the average precision of the model. (**c**) Relationship between model's predictive uncertainty and model's prediction error captured by the root mean squared error (RMSE). For each predicted standard deviation in the hold-out test data, find all test data with predicted standard deviations less than 0.05 away, and compute the corresponding averaged standard deviation and RMSE. Positive correlation indicate that model's prediction tends to be less accurate when the prediction uncertainty is high. We observe this overall trend across all models. For ensemble models, model uncertainties capture the agreement between different regressors. Higher standard deviation indicates less agreement between regressors. Source data are provided as a Source Data file.

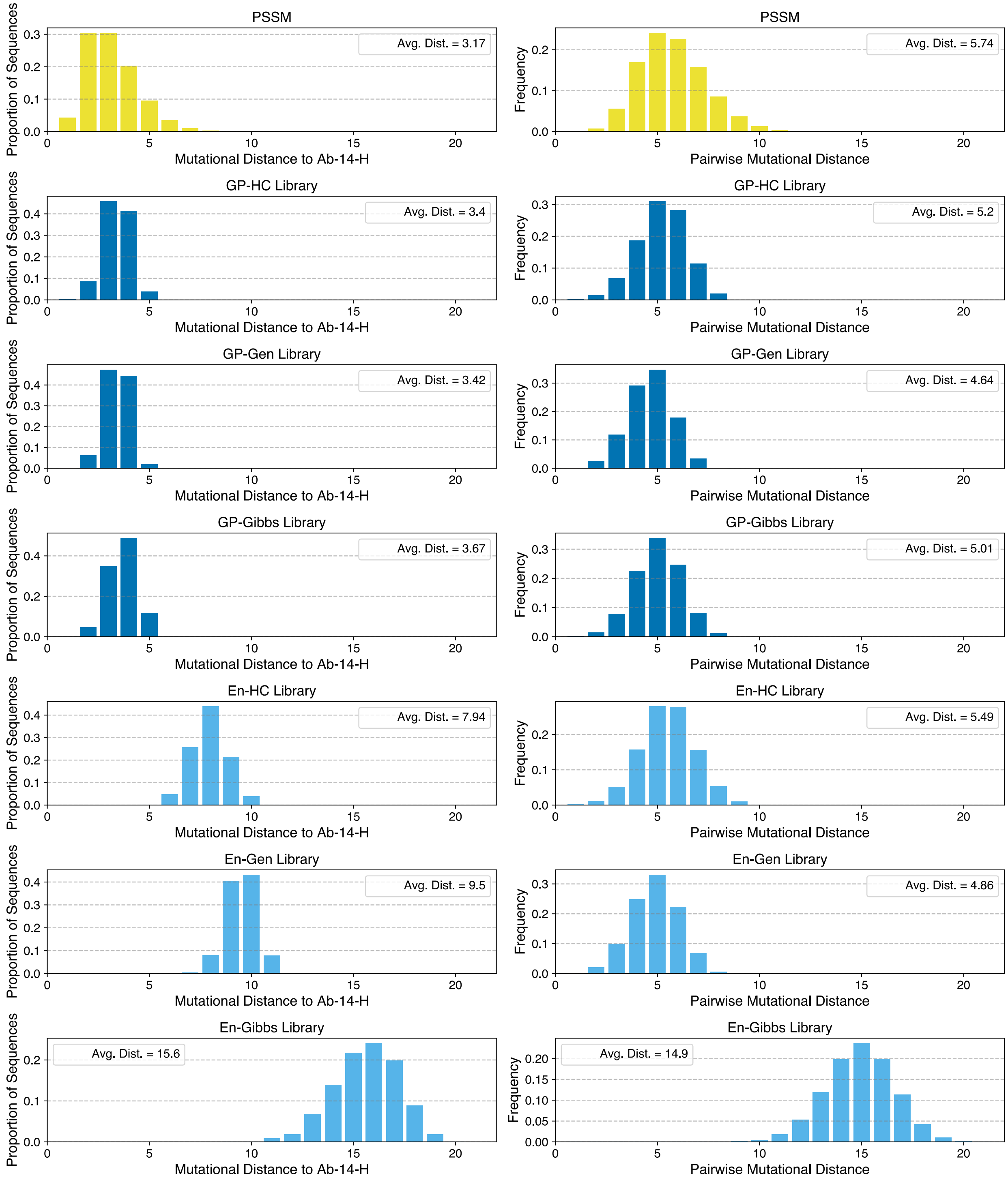

Supplementary Fig. 3 Distribution of diversity metrics by library for Ab-14-H variants. Source data are provided as a Source Data file.

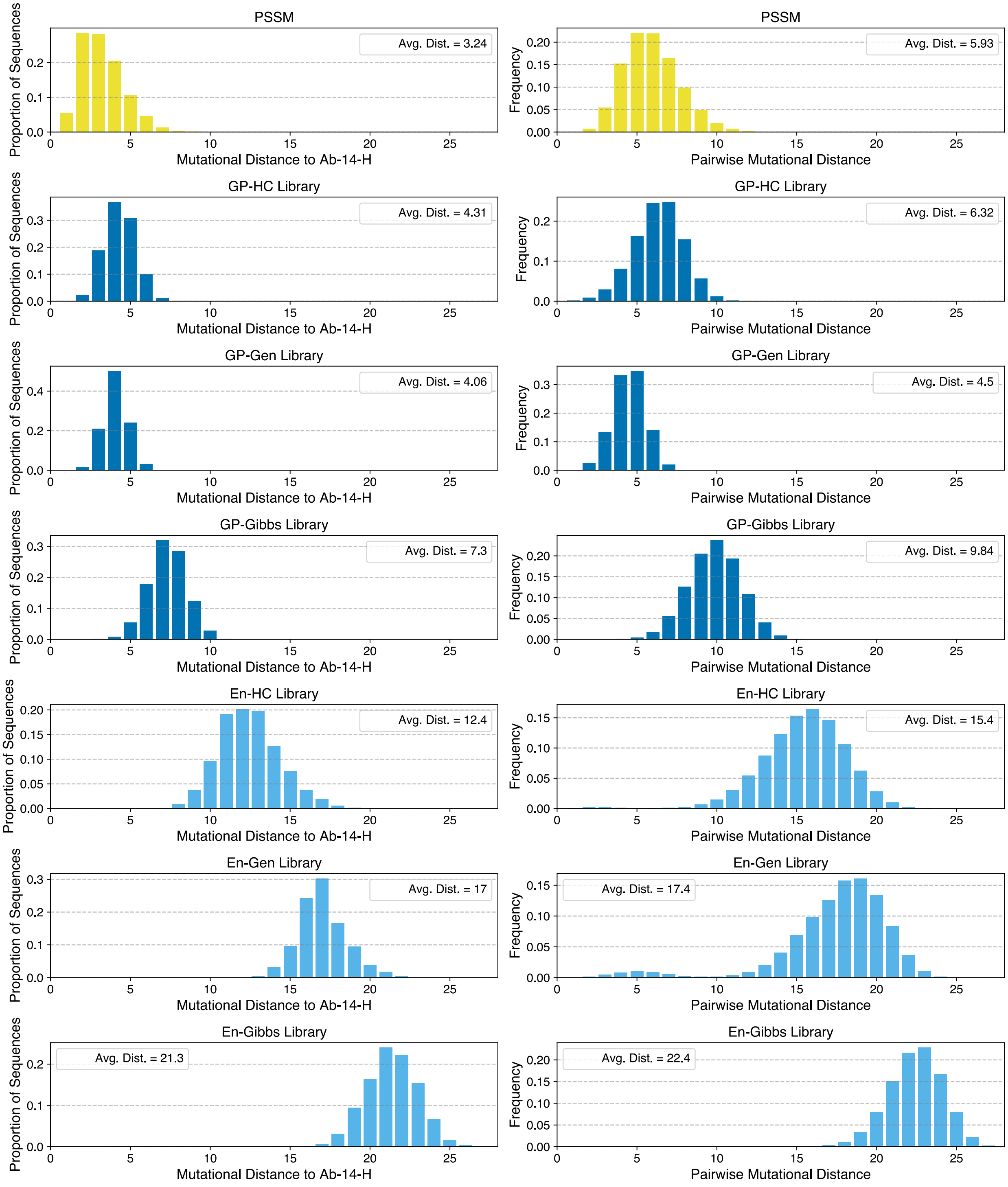

Supplementary Fig. 4 Distribution of diversity metrics by library for Ab-14-L variants. Source data are provided as a Source Data file.

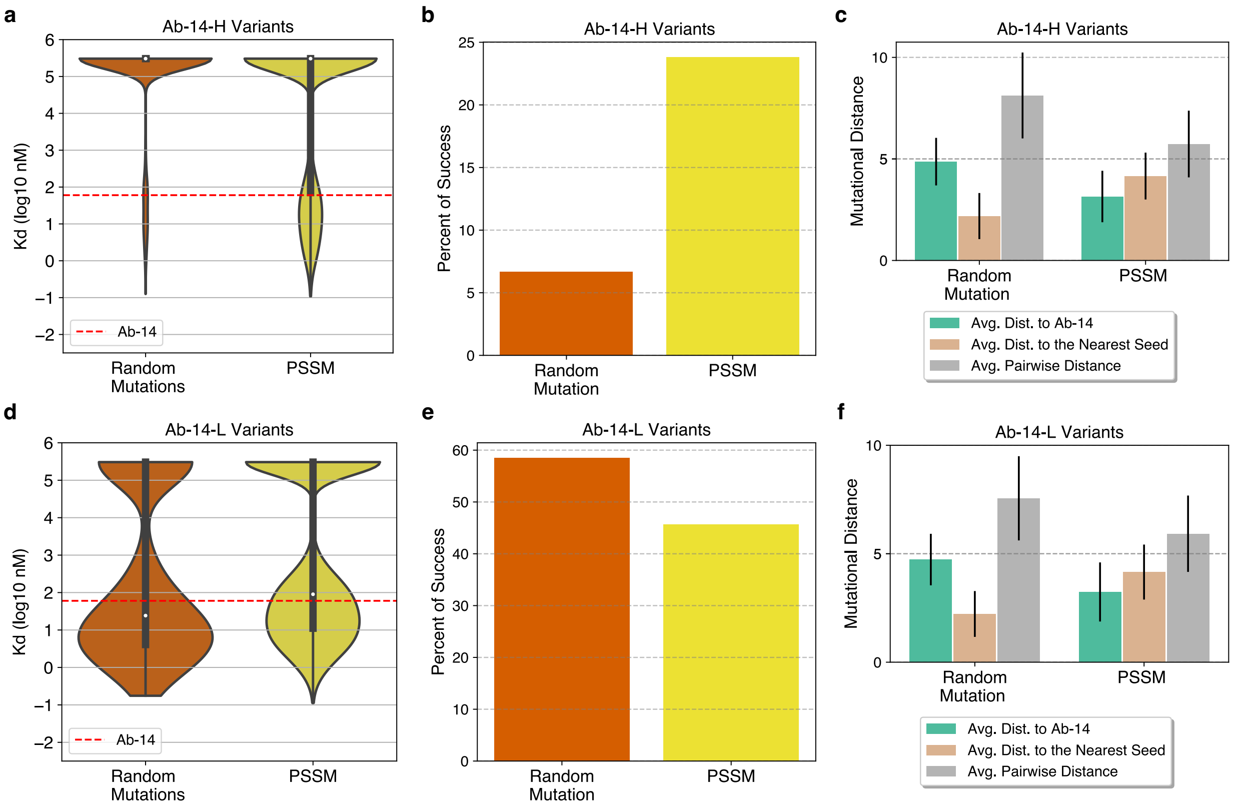

Supplementary Fig. 5 Random mutation library vs PSSM library. For sequences having at least 3 (out of 6) empirical binding affinities, we use the averaged values as ground-truth measured affinities. The rest of the sequences are considered as un-successful designs. All evaluations are performed over n=1616, 6510 Ab-14-H variant designs and n=465, 8188 Ab-14-L variant designs generated by random mutations and PSSM, respectively. (**a**) The violin plot is used to depict summary statistics and empirically measured affinity distribution of Ab-14-H heavy chain designs (center: median; limits: 1st and 3rd quartile; whiskers: +/- 1.5 IQR). Affinities of unsuccessful sequences are set to be 5.48 (the largest assay value of all Ab-14-H variants). (**b**) Percent of sequences that have stronger empirical binding affinity than the candidate antibody for all the Ab-14-H variant libraries. (**c**) Diversity comparison for all the Ab-14-H variant libraries. Data are presented as mean values and +/- standard deviation. The average distance to the nearest seed sequences is added to the comparison because random mutations are generated by randomly mutating the seed sequences. (**d**) The violin plot is used to depict summary statistics and empirically measured affinity distribution of Ab-14-L light chain designs (center: median; limits: 1st and 3rd quartile; whiskers: +/- 1.5 IQR). Affinities of unsuccessful sequences are set to be 5.53 (the largest assay value of all Ab-14-L variants). (**e**) Percent of success for all Ab-14-L variant libraries. (**f**) Diversity comparison for all the Ab-14-L variant libraries. Data are presented as mean values and +/- standard deviation. Source data are provided as a Source Data file.

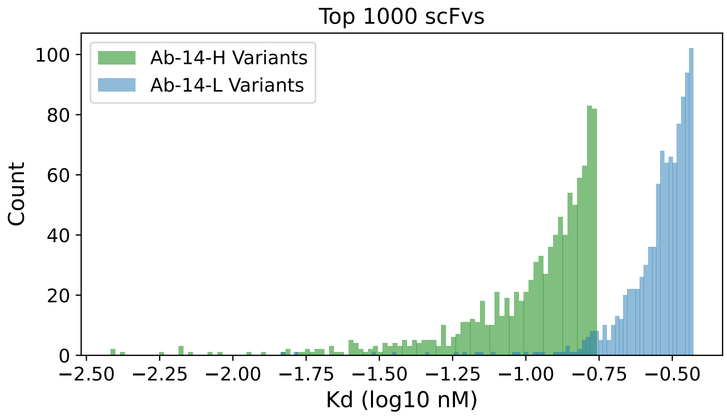

Supplementary Fig. 6 Heavy chain designs vs light chain designs. Histogram of top 1000 heavy chain and light chain designs over the empirically measured affinities; heavy chain designs led to significantly stronger binders (lower Kd values) than light chain designs. Source data are provided as a Source Data file.

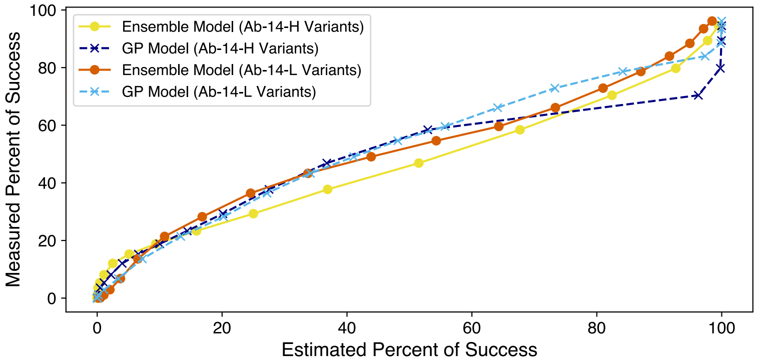

Supplementary Fig. 7 Evaluation of proposed in silico metric for library performance prediction. The estimated percent of success aligns well with the true performance on the hold-out test data. Source data are provided as a Source Data file.

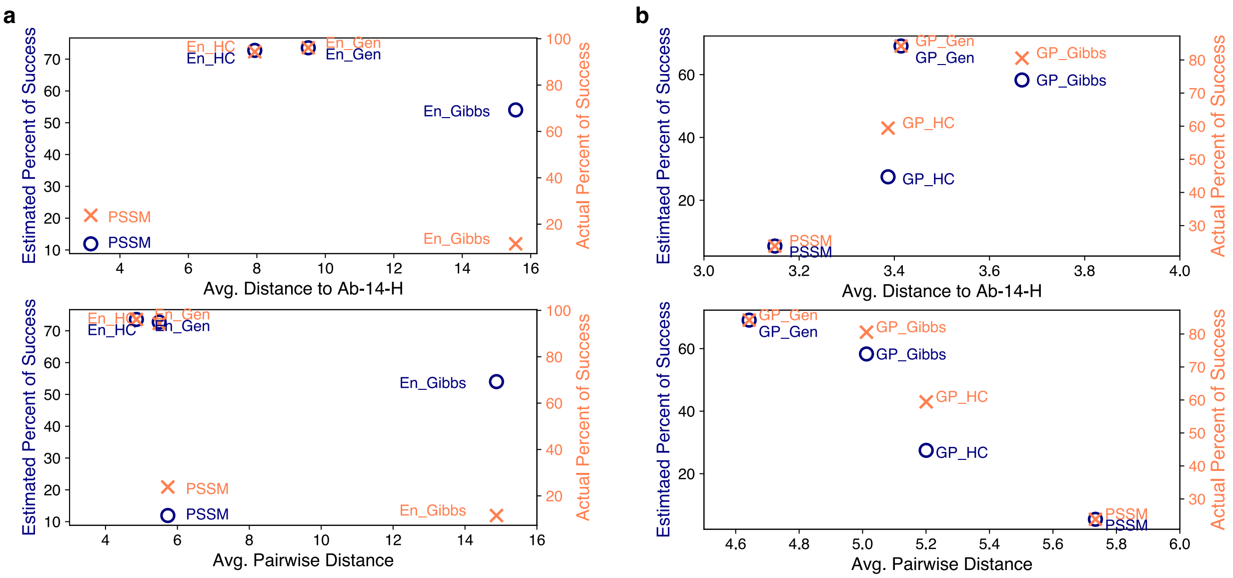

Supplementary Fig. 8 The estimated percent of success metric enables exploration of the tradeoffs between performance and diversity and informs library selection (Ab-14-H variant designs). The relative performance of the estimated percent of success matches well to the empirically measured percent of success. (**a**) ensemble-based libraries for Ab-14-H variants. (**b**) GP-based libraries for Ab-14-H variants. Source data are provided as a Source Data file.

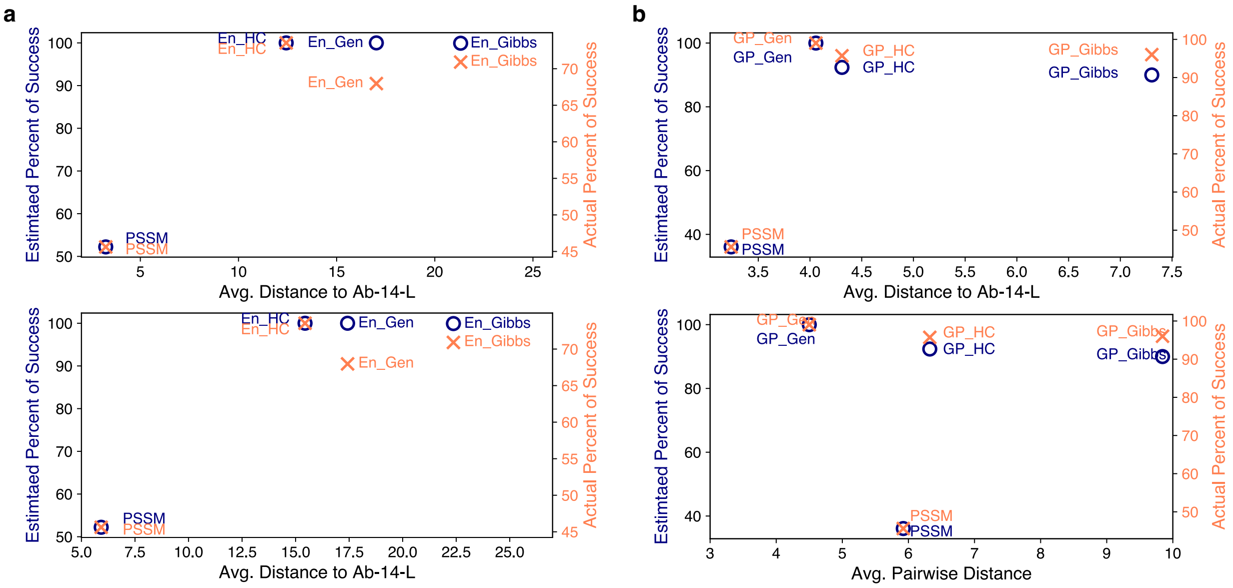

Supplementary Fig. 9 The estimated percent of success metric enables exploration of the tradeoffs between performance and diversity and informs library selection (Ab-14-L variant designs). The relative performance of the estimated percent of success matches well to the empirically measured percent of success. (**a**) ensemble-based libraries for Ab-14-L variants. (**b**) GP-based libraries for Ab-14-L variants. Source data are provided as a Source Data file.

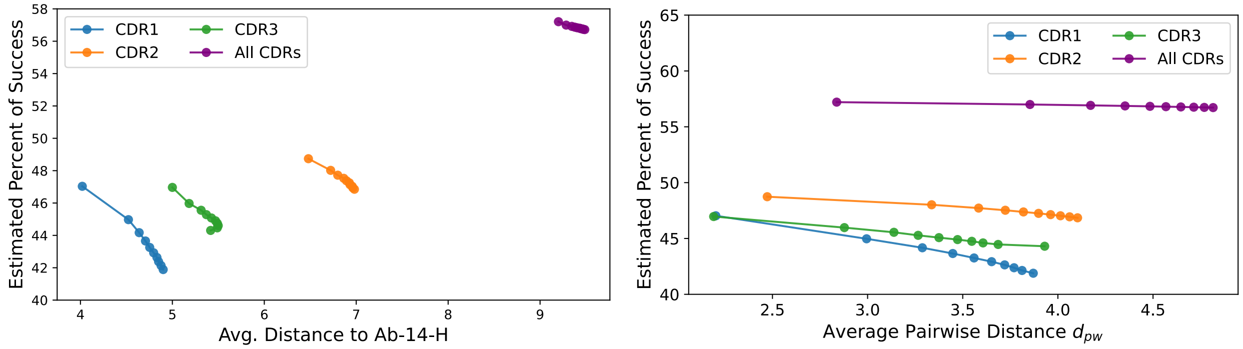

Supplementary Fig. 10 Evaluation of designing various antibody CDR regions. Library comparison on designing the entire heavy chain CDRs and individual CDRs as the number of sequences in each library increases. Designing the entire heavy chain CDRs produces antibodies with higher estimated percent of success and diversity. Source data are provided as a Source Data file.

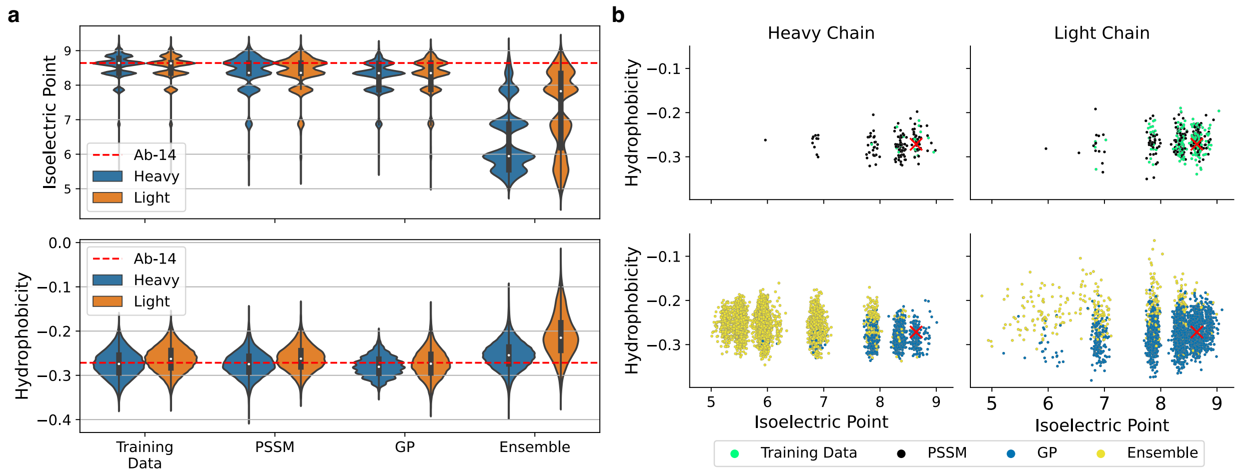

Supplementary Fig. 11 Biophysical and sequence properties of heavy and light chain libraries. (**a**) Violin plots of isoelectric points (pI) and hydrophilicities of heavy and light chain sequences in the training data, and PSSM-, GP-, and ensemble-designed libraries (center: median; limits: 1st and 3rd quartile; whiskers: +/- 1.5 IQR). Evaluations are performed over n = 26454, 6510, 12407, 14835 Ab-14-H variants and n = 26224, 8188, 17500, 17872 Ab-14-L variants from the Training data, PSSM-, GP-, and ensemble-generated libraries, respectively. The dashed lines represent the value of the corresponding candidate Ab-14. The pI values calculated for most of the Ab-14-H and Ab-14-L variants are in the 7.5-9.0 interval. The exception is in the ensemble-based method, in which it exhibits a wider pI value range (5.0-9.0), and many Ab-14-H variants have acidic pI (below 6.5). Similarly, the hydrophobicity values of ensemble-based libraries also have a wider value range. (**b**) Scatter plots showing the joint distribution of pI and hydrophilicities for sequences with strong binding affinity (measured binding affinity <= 1 nM). The top row shows the results for the training data and PSSM libraries; the bottom row shows the GP and ensemble libraries. The 'x' marker indicates isoelectric point and hydrophobicity of Ab-14. Our designed strong binders cover a wide range of these biophysical properties. Source data are provided as a Source Data file.

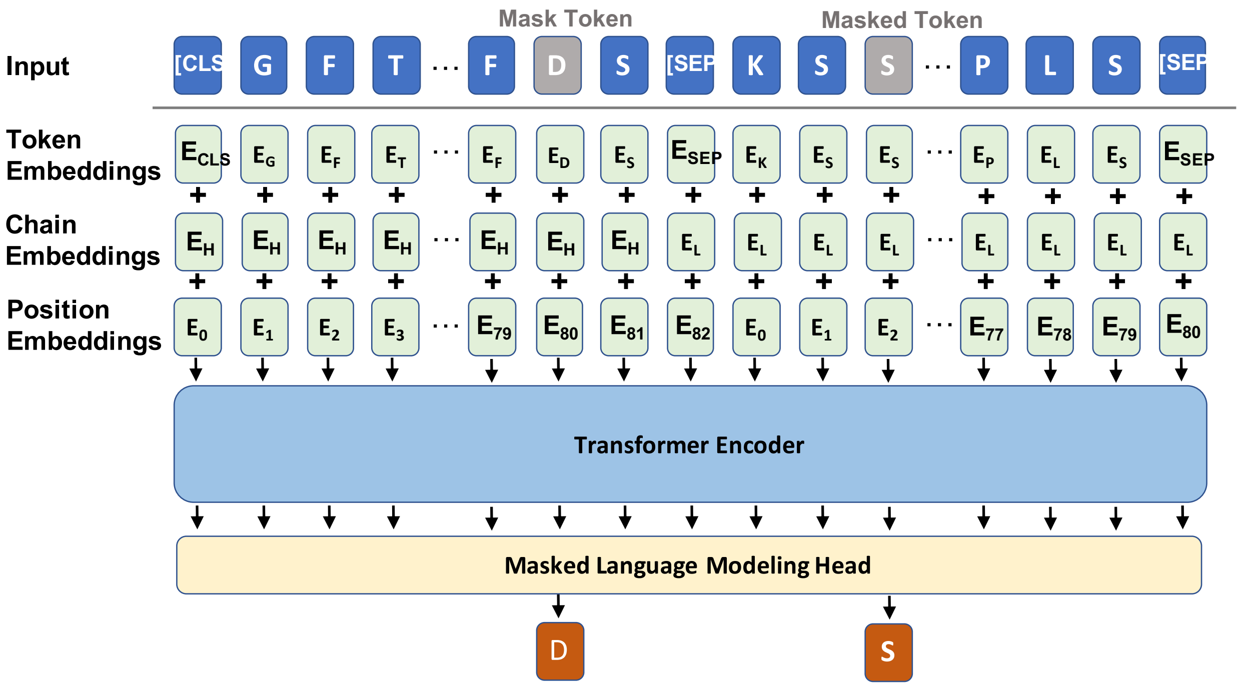

Supplementary Fig. 12 Masked language modeling with BERT transformer. For training the protein language model, heavy-chain antibody language model and light-chain antibody language model, the input to the model only consists of the part before the first [SEP] token. For training the paired heavy-light chain language model, the chain embedding and position embedding are encoded based on the specific chain the token belongs to and its integer index in the corresponding chain.
